# Tools of the trade: estimating time-varying connectivity patterns from fMRI data

**DOI:** 10.1093/scan/nsaa114

**Published:** 2020-08-12

**Authors:** Armin Iraji, Ashkan Faghiri, Noah Lewis, Zening Fu, Srinivas Rachakonda, Vince D Calhoun

**Affiliations:** Tri-Institutional Center for Translational Research in Neuroimaging and Data Science (TReNDS), Georgia State University, Georgia Institute of Technology, and Emory University, Atlanta, GA 30303, USA; Tri-Institutional Center for Translational Research in Neuroimaging and Data Science (TReNDS), Georgia State University, Georgia Institute of Technology, and Emory University, Atlanta, GA 30303, USA; Tri-Institutional Center for Translational Research in Neuroimaging and Data Science (TReNDS), Georgia State University, Georgia Institute of Technology, and Emory University, Atlanta, GA 30303, USA; Tri-Institutional Center for Translational Research in Neuroimaging and Data Science (TReNDS), Georgia State University, Georgia Institute of Technology, and Emory University, Atlanta, GA 30303, USA; Tri-Institutional Center for Translational Research in Neuroimaging and Data Science (TReNDS), Georgia State University, Georgia Institute of Technology, and Emory University, Atlanta, GA 30303, USA; Tri-Institutional Center for Translational Research in Neuroimaging and Data Science (TReNDS), Georgia State University, Georgia Institute of Technology, and Emory University, Atlanta, GA 30303, USA

**Keywords:** dynamic functional connectivity, spatially dynamic, temporally dynamic, spatiotemporally dynamic, fMRI

## Abstract

Given the dynamic nature of the brain, there has always been a motivation to move beyond ‘static’ functional connectivity, which characterizes functional interactions over an extended period of time. Progress in data acquisition and advances in analytical neuroimaging methods now allow us to assess the whole brain’s dynamic functional connectivity (dFC) and its network-based analog, dynamic functional network connectivity at the macroscale (mm) using fMRI. This has resulted in the rapid growth of analytical approaches, some of which are very complex, requiring technical expertise that could daunt researchers and neuroscientists. Meanwhile, making real progress toward understanding the association between brain dynamism and brain disorders can only be achieved through research conducted by domain experts, such as neuroscientists and psychiatrists. This article aims to provide a gentle introduction to the application of dFC. We first explain what dFC is and the circumstances under which it can be used. Next, we review two major categories of analytical approaches to capture dFC. We discuss caveats and considerations in dFC analysis. Finally, we walk readers through an openly accessible toolbox to capture dFC properties and briefly review some of the dynamic metrics calculated using this toolbox.

## Dynamic functional connectivity (time-varying functional patterns)

### Introduction and definitions

It has been suggested that cognition and many mental activities result from the interactions of distributed brain areas (Bressler and Menon, 2010). A local neural assembly, which has its own intrinsic functionality, interacts at the global level with other parts of the brain. However, a major challenge to studying the brain from this point of view is how to best capture functional interactions across the whole brain. An ideal solution would be to evaluate whole-brain dynamic interactions at the neural level; however, imaging at such a scale in humans is not possible at present. Instead, functional imaging techniques can be used to assess the whole-brain functional interactions at a macroscale (mm) resolution and have yielded information of great value.

Functional magnetic resonance imaging (fMRI) measures the blood oxygenation level-dependent (BOLD) signal, a macroscale proxy for average neural activity, which allows simultaneous investigation of the functional localization and interactions between brain regions. Most commonly, the entire fMRI scan is used to calculate the average functional connectivity (FC), a method known as static functional connectivity (sFC). However, spontaneous brain activity is rich with dynamic properties which are disregarded in such a method. Therefore, research into whole-brain dynamic functional connectivity (dFC) has become a burgeoning field of study since initial work on the topic (Sakoglu *et al.*, 2010). Although one can distinguish between approaches that leverage the time-varying signal and those that more explicitly model/capture dynamics over time (Lurie *et al.*, 2020), we refer to both as dFC for convenience. dFC is defined as time-varying FC and contains information regarding the temporal reconfiguration of functional entities (also called sources). dFC studies aim to evaluate how the interactions between functional sources change over time. We can define a functional source as a group of temporally synchronized neural assemblies, which present similar functionality within a given dataset (Iraji *et al.*, 2020). Fixed anatomical locations are convenient representations of these sources, assuming all voxels within a predefined anatomical location have the same functional profile and are the same across individuals. More advanced approaches, such as dynamic functional network connectivity (dFNC), leverage the data itself to estimate the sources and study dFC (Jafri *et al.*, 2008; Allen *et al.*, 2011; Calhoun and de Lacy, 2017).

### Potential relationship with brain function and neural activity

It should be noted that because fMRI is an indirect measurement of neural activity, fluctuations in FC estimated by fMRI are also indirect representations of dFC. There is ongoing discussion regarding how well these fluctuations capture the underlying brain dynamism, but previous studies provide a significant amount of evidence to support the potential relationship between the fluctuations in FC obtained from fMRI and neural dynamics in the brain (for review, see (Lurie *et al.*, 2020)). For instance, simultaneous fMRI and electroencephalography (EEG) imaging studies show that fluctuations in FC obtained from resting-state fMRI are associated with electrophysiological signatures of EEG (Chang *et al.*, 2013; Allen *et al.*, 2018). A comparison between the hemodynamic signal and neuronal calcium signal also provides strong evidence that temporal fluctuations in hemodynamic FC are related to brain dynamics (Matsui *et al.*, 2019). Thus, to simplify notation in the remainder of this article, we refer to time-varying FC estimates from fMRI as dFC.

### Evidence of reliability and cognitive relevance

Studies have also evaluated the replicability and reliability of dFC properties captured by fMRI. Previous researchers used a large dataset (∼7500 subjects) collected from different sites and identified replicable dFC patterns, which are robust against variations in data quality and analysis methods (Abrol *et al.*, 2017). Test–retest reliability analyses show the presence of reliable dynamic patterns (Choe *et al.*, 2017; Zhang *et al.*, 2018a). Similar studies also identify reliable and reproducible intersession (intervals of 2 days and 1 week) dFC patterns (Yang *et al.*, 2014; Smith *et al.*, 2018). Shi *et al.* (2018) reported robust findings of the association between dFC and subjective well-being across two independent datasets and different analysis parameters, which suggest dFC is involved in self-focused processing, emotional regulation and the cognitive control process.

dFC also helps to index mental states dictated by a multitask paradigm and can differentiate between task-induced cognitive processes (Gonzalez-Castillo *et al.*, 2015). dFC patterns of the salience network and the posteromedial cortex are related to individual differences in cognitive flexibility and categorization ability (Yang *et al.*, 2014; Chen *et al.*, 2016). Using a continuous auditory detection task, Sadaghiani *et al.* (2015) showed that dFC features before the auditory stimulus could predict whether the audio was recognized or not, and dFC patterns after stimulation were also significantly different between the two scenarios. Madhyastha *et al.* (2015) show that dFC within the dorsal attention network and frontoparietal network (FPN) predicts attention task performance. The dFC variability (see Table 1 for the definition of different dFC measures) between the default mode network (DMN) and FPN is shown to be related to cognitive performance (Douw *et al.*, 2016). Compared to low trait mindfulness individuals, high trait mindfulness individuals show a higher level of transitions between brain dFC states and spend more time in one dFC state associated with task-readiness (Lim *et al.*, 2018). Marusak *et al.* (2018) similarly found that dFC (but not sFC) is related to mindfulness in youths such that more mindful youths have a higher level of transitions between dFC states. Additionally, Cabral *et al.* (2017) report a relationship between cognitive performance in older adults and slow switching between dFC states. dFC properties appear to be related to age in the early years as well.

**Table 1.** List of dFC measures

For stateless dynamic measures:
dFC variability: It represents the amount of variation in dFC over time and is commonly calculated as the standard deviation of dFC value across time/time windows. Coupling variability map: It is the spatial map of the amount of variation in the dFC of a given source/network over time, and it is estimated by calculating voxel-wise changes in the dFC of the source using the L1 norm distance (sum of absolute differences). Spatiotemporal transition matrix: It summarizes the whole brain dFC of a given source into a matrix in which each element of the matrix is the number of times that dFC value changes from one FC range to another. Several global metrics can be estimated from the spatiotemporal transition matrix including energy, entropy and homogeneity.
For state-based dynamic measures:
dFC strength: The strength of FC in a given state. Dwell time: The average amount of time that a subject lingers in each state. Occupancy rate: The percentage of time that each state occurs during a scan. Transition matrix: The probability of transitioning from one state to another. Average variability index: It is an indication of the overall level of dynamism for a functional source. Variability index is defined as the standard deviation of the binomial distribution and estimates the level of variability in a region’s association to a given source. Functional (inter-domain) state connectivity: It captures the level of concurrency between states of different sources (e.g. functional domains) when a technique (e.g. spatial dynamic hierarchy) estimates dynamic states for each source separately.
For meta-state dynamic measures:
Number of realized meta-states: the number of distinct meta-states that an individual realizes during the length of a scan. Meta-state switching: the number of times an individual switches from one meta-state to a different meta-state during the length of a scan. Meta-state span: Maximally different (in the L1 sense) meta-states that a given subject realizes. Meta-state total trajectory length: Total distance traveling in the state space which is the sum of L1 distances between successive meta-states for each subject. Level-*k* hub meta-states: the meta-states that an individual visit at least *k* times during the scan. Level-*k* transient meta-states: the meta-states that an individual visits less than *k* times during the scan.

Interestingly, regions with different cognitive and processing demands represent different levels of dynamism. The brain networks/areas that are known to be involved in higher cognitive processing show a higher level of dynamism measured by dFC than networks/areas engaged in primary processing (Zalesky *et al.*, 2014; Chen *et al.*, 2016; Iraji *et al.*, 2019b). At the network level, networks that are engaged in a wide range of cognitive functions, such as the FPN, seem to have the highest level of dynamism (Zalesky *et al.*, 2014; Iraji *et al.*, 2019b). The relevance of dFC is also supported by other studies that evaluate the relationship between dFC properties and biological features such as age, gender and cognitive processes (Thompson *et al.*, 2013a; Leonardi *et al.*, 2014; Elton and Gao, 2015; Hutchison and Morton, 2015; Qin *et al.*, 2015; Yaesoubi *et al.*, 2015a; Yaesoubi *et al.*, 2015b; Shine *et al.*, 2016a; Shine *et al.*, 2016b; Kucyi *et al.*, 2017; Cai *et al.*, 2018; Kucyi, 2018).

The findings mentioned earlier support the potential neurophysiological relevance of dFC and the benefit of studying dFC properties to elucidate the brain function. One key future research direction is the use of cognitive/affective tasks to determine the specific dFC properties of networks/areas which are associated with certain cognitive processing and mental states.

### Neurological and mental disorders

dFC analysis also has the potential to improve the understanding of impaired brains and the functional alterations caused by brain disorders. Compared to static analysis, dFC analysis provides additional information about the temporal profile of brain function and its changes in disorders. dFC analysis can detect nuanced alterations that are averaged out in static analysis (Calhoun *et al.*, 2014; Iraji *et al.*, 2019a). In addition, dFC has been shown to explain some of the inconsistencies in sFC findings (Iraji *et al.*, 2019a). dFC studies also suggest promising research directions in neurological and psychiatric disorders (see ‘Considerations and caveats’ for further details) (Calhoun *et al.*, 2014). Towards this goal, researchers have started using different methods and metrics to investigate the characteristics of dFC in various brain disorders.

Among various brain disorders, schizophrenia (SZ) has been one of the most widely studied via dFC. Patients with SZ spend less time in a dFC state with strong connectivity, and the strengths of dFC between subcortical and sensory networks are weaker in these patients (Damaraju *et al.*, 2014). Spatial dynamics studies have revealed weaker dFC strength (transient hypoconnectivity) within particular networks (Iraji *et al.*, 2019a; Iraji *et al.*, 2019b) in SZ. It has been shown that the decrease in dFC strength is accompanied by higher fluctuations in dFC between brain regions (Yue *et al.*, 2018) and within and between certain brain networks (Ma *et al.*, 2014; Iraji *et al.*, 2019a) in patients with SZ. Studies also show frequency-specific dFC alterations in patients with SZ (Yaesoubi *et al.*, 2017; Zhang *et al.*, 2018b; Faghiri *et al.*, 2019). The atypical dFC patterns in patients with SZ may be related to disease traits (Fu *et al.*, 2018; Yue *et al.*, 2018; Iraji *et al.*, 2019a).

In another clinical condition, a study found that individuals with major depressive disorder (MDD) spend more time in a state with weak FC strength across the brain (weakly connected dFC state) associated with self-focused thinking (Zhi *et al.*, 2018). Atypical dFC patterns were significantly correlated with both depressive symptoms and cognitive performance (Zhi *et al.*, 2018). Qiu *et al.* (2018) studied the dFC of the amygdala’s subregions in untreated individuals with first-episode MDD and found a decrease in the dFC strength between specific amygdala subregions and several regions within the limbic–cortical–striato–pallido–thalamic circuit. Additionally, they showed that the age of MDD onset correlates with the dFC strength between the left centromedial subregion of the amygdala and the brainstem (Qiu *et al.*, 2018). In a different study, MDD patients show decreased variability of dFC between the DMN and the PFN (Demirtas *et al.*, 2016).

Alterations in dFC features have been observed in patients with dementia as well. Change in anterior-posterior dFC of the brain was associated with declined episodic memory performance in the elderly (Quevenco *et al.*, 2017). Patients with Alzheimer’s disease (AD) showed higher and lower dwell time in dFC state with strong the anterior and posterior DMN, respectively (Jones *et al.*, 2012). Patients with AD also show alteration in whole-brain dFC (Cordova-Palomera *et al.*, 2017; Schumacher *et al.*, 2019) and spend more time in the weakly connected dFC state (Schumacher *et al.*, 2019). Patients with AD show both common and distinct dFC patterns with patients who have subcortical ischemic vascular disease, while the clinical features and symptoms between these two disorders can sometimes be difficult to distinguish (Fu *et al.*, 2019a).

In autism spectrum disorder (ASD), alterations in dFC patterns were observed both within the DMN and between the DMN and networks involved in higher cognitive processing, including the PFN and the cingulo-opercular network (de Lacy *et al.*, 2017; Rashid *et al.*, 2018). Fu *et al.* (2019b) found that individuals with ASD show a transient increase in dFC strength between hypothalamus/subthalamus and sensory networks, as well as alterations in several meta-state metrics including the number of meta-states and total traveling distance. Interestingly, these atypical dFC patterns are significantly associated with the total autism diagnostic observation schedule score. Individuals with ASD show a decrease in dFC strength between the right anterior insula and the regions associated with the DMN, including the ventral medial prefrontal cortex and posterior cingulate cortex (PCC) dFC (Guo *et al.*, 2019). It has been suggested that decreased dFC variability of the PCC is related to the role of the DMN in the social-cognitive deficits of ASD. The lower dFC variability between the PCC and sensorimotor cortex was correlated with deficits in social motivation and social relating in ASD individuals (He *et al.*, 2018). Harlalka *et al.* (2019) reported positive correlations between autism diagnostic observation schedule scores and dFC variability, particularly in the DMN connections.

The dFC of the DMN and its associated areas were also associated with cognition in other populations. In (left) temporal lobe epilepsy, the lower dFC variability of the PCC was related to disturbed verbal memory functioning (Douw *et al.*, 2015). The dFC of the DMN and memory functioning were positively correlated in Parkinson’s disease patients (Engels *et al.*, 2018). Parkinson’s disease patients also show a loss of specificity of dFC in putaminal subunits with the exception of the caudal middle frontal gyrus (Liu *et al.*, 2018). Changes in the dFC strength of the putamen subunits (particularly anterior subunits) are shown to be correlated with the unified Parkinson’s disease rating scale III (UPDRS III), and joint dFC features (strength and variability) were able to predict UPDRS III and Montreal cognitive assessment scores (Liu *et al.*, 2018).

Alterations in dFC states were also observed in mild traumatic brain injury patients (Vergara *et al.*, 2018; Hou *et al.*, 2019; van der Horn *et al.*, 2019) and had better discriminatory power than sFC (Vergara *et al.*, 2018). In relapsing-remitting multiple sclerosis, dorsal and ventral attention networks displayed lower within-network dFC and higher between-network dFC, and the dFC alterations were linked to white matter lesion damage (Huang *et al.*, 2019). Better executive functions in relapsing-remitting multiple sclerosis patients were associated with higher dFC (Lin *et al.*, 2018). Atypical dFC patterns present in other cohorts and brain disorders, including migraine (Tu *et al.*, 2019), stroke (Chen *et al.*, 2018), epilepsy (Douw *et al.*, 2015; Liu *et al.*, 2017; Klugah-Brown *et al.*, 2019), attention deficit hyperactivity disorder (Ou *et al.*, 2014; de Lacy and Calhoun, 2019), post-traumatic stress disorder (Li *et al.*, 2014; Jin *et al.*, 2017), frontotemporal dementia (Premi *et al.*, 2019) and Lewy body dementia (Schumacher *et al.*, 2019).

Despite the rising interest and great potential of dFC in various circumstances, the application of dFC is still not a baseline tool for neuroscientists. One main reason is the rapidly growing pool of analytical approaches. In the following sections, we provide a basic summary of the most common analytical approaches and a straightforward tool to study dFC.

## Analytic approaches to study dFC

**Fig. 1.**
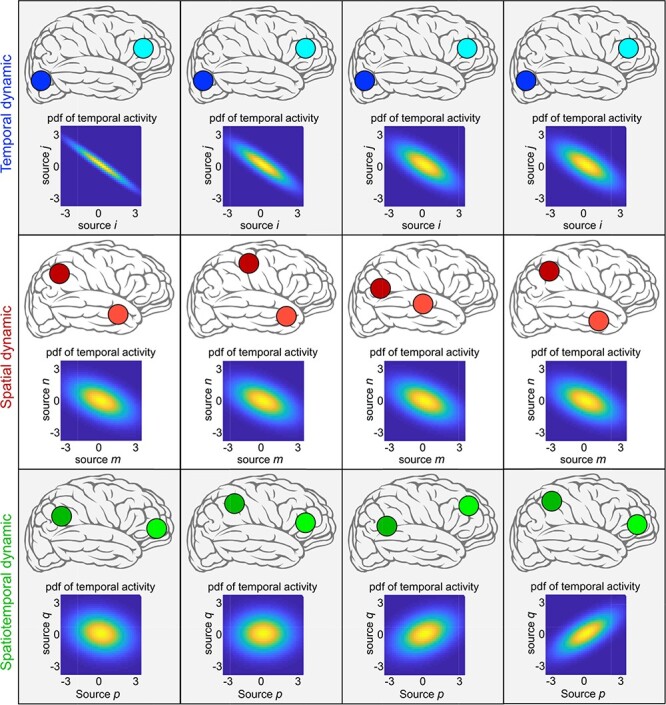
Example of temporal, spatial and spatiotemporal dynamic with a scenario that the brain has only two functional sources. The brain is temporally dynamic if the temporal coupling between the temporal activity of sources varies over time. The brain is spatially dynamic if the spatial properties of sources change over time (e.g. translations of sources in space). If functional sources hold both spatially and temporally dynamic properties, it is spatiotemporally dynamic.

**Fig. 2.**
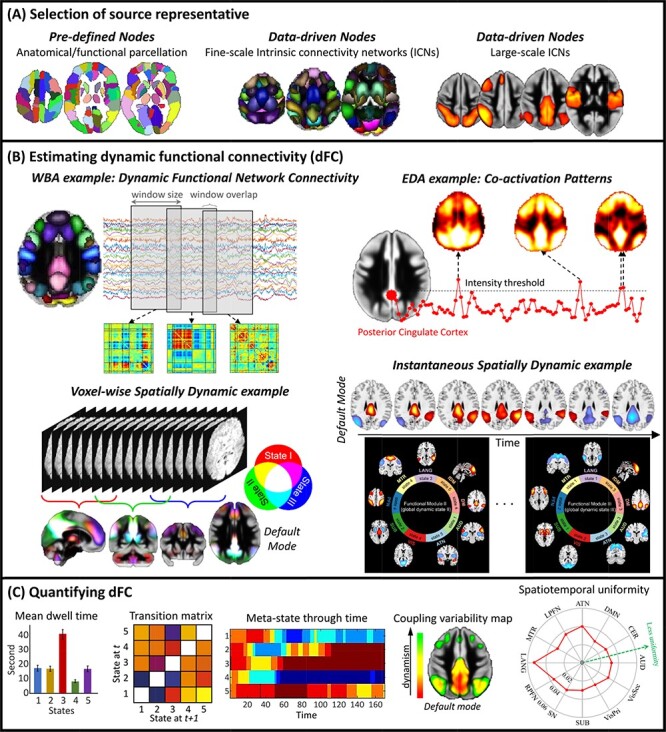
Cartoon examples of the analysis pipeline dFC analysis. (A) We first select nodes (proxies for spatial locations of sources) to calculate functional connectivity between sources of interests. (B) We use a dFC estimator approach to calculate dFC between selected nodes. Different estimators measure different dynamic properties (temporal dynamic or spatial dynamic). WBA: Window-based approaches, EDA: Event detection approaches. (C) After estimating dFC, various techniques can be used to quantify dFC and evaluate dynamic properties.

In general, dFC studies probe the dynamic properties of the brain via variations in FC estimations over time. A dFC study can evaluate changes in spatial patterns of functional sources over time (also known as spatial dynamics) and/or variations in their activity profiles of sources over time (which is called temporal dynamics) (Figure 1) (Iraji *et al.*, 2020). Several analytical techniques have been proposed to capture and evaluate dFC using fMRI data (Figure 2). Here, we review essential concepts and terms behind two major non-exclusive categories, ‘window-based approaches (WBAs)’ and ‘event detection approaches (EDAs)’. There are several technical reviews on different approaches of these two categories, as well as other categories of methods, such as those that use dynamic modeling techniques or temporal sequence information (Hutchison *et al.*, 2013; Calhoun *et al.*, 2014; Keilholz *et al.*, 2017; Preti *et al.*, 2017).

FC is estimated by calculating the statistical association between measured brain signals, commonly between different spatial localities (nodes) (Figure 2A). A node can be a voxel, an anatomical region/seed or computed from the fMRI data itself, e.g. an intrinsic connectivity network (ICN). Functional homogeneity within a node is the crucial factor in defining nodes. For instance, when we use anatomical regions as nodes, we should verify that the voxels within a node must have more similar time courses than voxels from different nodes. To ensure functional homogeneity, we can use data-driven approaches like independent component analysis (ICA) to estimate the ICNs as nodes (Calhoun and Adali, 2012). ICA is a multivariate approach that simultaneously estimates the spatial patterns and activity profiles (time courses) of ICNs. For simplicity, we assume FC is calculated using Pearson correlation, but other metrics such as coherence are equally valid and can be used to capture additional information (Yaesoubi *et al.*, 2015a; Salman *et al.*, 2019).

### Window-based approaches

The substantial similarity with conventional FC (i.e. sFC) and ease of use make the applications of WBAs to study dFC and the interpretation of their findings straightforward. Window-based approaches (commonly known as sliding-window approaches), in simple terms, estimate conventional FC for durations larger than the sampling rate of the acquired data (Sakoglu *et al.*, 2010). The time courses are divided into short segments (time windows), and FC is calculated for each time window. This results in a series of windowed-FC over time (FC as a function of time) that contains dFC information. WBAs require defining time window and the node(s) of interest before calculating windowed-FC (Figure 2B).

Time windows can have different sizes, shapes and window overlaps, but these parameters commonly remain constant throughout a study. The best choice of these parameters is unknown and can be different depending on the available data and the goals of studies. However, we can make a general recommendation from previous literature. For the window shape, the most common choice is tapered windows (Allen *et al.*, 2014; Leonardi and Van De Ville, 2015). For the window overlap, windows with zero overlap or and maximum overlap (all time points except one time point are shared between consecutive windows) are the two most common choices. The most important aspect of time windows to capture dFC is the window size. Very small window sizes may not have enough information to estimate dFC robustly and thus introduce spurious fluctuations while large window sizes may smooth out the dynamic properties and fail to capture dynamic properties (Fu *et al.*, 2014; Vergara *et al.*, 2019). Ideally, we should choose window sizes that match the timescale of underlying brain dynamism; however, there is no prior information about the underlying dFC profile. At the same time, studies suggest WBAs can distinguish underlying mental states even using window sizes substantially different from the duration of the underlying cognitive processes. For instance, Gonzalez-Castillo *et al*. (2015) modulate mental states using a series of well-defined cognitive tasks. While the optimal window size to match the transition between mental states was 180 s, window sizes as short as 22.5 s accurately track mental states. A recommended window size is between 30 and 60 s (Leonardi and Van De Ville, 2015; Zalesky and Breakspear, 2015).

Meanwhile, several approaches have been proposed to circumvent the selection of window size. One may use adaptive window size approaches to match window size with the underlying brain dynamics by estimating local stationarity or change points (Fu *et al.*, 2014; Xu and Lindquist, 2015; Jeong *et al.*, 2016; Jin *et al.*, 2017). One can also explore dFC at different frequencies which is similar to adapting the window size to the frequency scale (Chang and Glover, 2010; Yaesoubi *et al.*, 2015a). Another solution is to estimate FC for single time points (instantaneous) FC and reduce or even eliminate the need of choosing a window (Thompson *et al.*, 2018; Yaesoubi *et al.*, 2018; Faghiri *et al.*, 2019, 2020; Iraji *et al.*, 2019b). The next step after defining our window parameters is to select the node(s) of interest. Commonly, we select the nodes from across the whole brain to summarize the whole-brain dFC as per-window FC matrices for the given nodes (Allen *et al.*, 2014). We can also focus on the dFC of a specific set of nodes based on the hypothesis of a study (Yang *et al.*, 2014). Another option is to calculate the FC of each source with every voxel of the brain for every time window to provide a detailed whole-brain, comprehensive map of the dFC of each source (Iraji *et al.*, 2019a).

Once dFC is calculated via WBAs, various techniques can be used to quantify dFC and evaluate dynamic properties (Figure 2C) (see ‘Implementing a dFC study: a GIFT walkthrough’ for some examples of metrics to quantify dFC properties). One standard procedure is to determine distinct and recurring dynamic patterns such as dFC (meta-)states and calculate dFC properties by assessing the temporal profiles of (meta-)states. dFC states are a set of distinct FC patterns, which are commonly identified by grouping windowed-FC using k-means clustering. In this way, each window is assigned to one state (Allen *et al.*, 2014). Meta-state analysis, on the other hand, assumes windowed-FCs are a combination of meta-states with continuous contributions over time (Miller *et al.*, 2016). Indeed, there is a close relationship between states and meta-states. Meta-states are equal to states if only one of them is present at any given time (power of all but one of them is zero). It is worth mentioning that both terms, states and meta-states, have been used for different purposes in literature. For instance, while in dFC studies, meta-state refers to an instantaneous coordinate of the brain in the state-space (Miller *et al.*, 2016), it has also been used to refer to FC patterns which recur across sessions (Shine *et al.*, 2016b). As the actual number of (meta-) states of the brain is unknown, it needs to be estimated using different techniques and criteria. Event detection approaches (see ‘Event detection approaches’) carry the same limitations as they estimate dynamic states in a similar manner. (Meta-) states can then be evaluated by different metrics such as dwell time, fraction rate, the number of transitions (switching), total traveling distance, the total number of (meta-) states met during the length of scan and community/modularity of states. We can also directly evaluate the dFC properties from windowed-FC, for instance, by calculating FC variation over time (dFC variability) and spatiotemporal transition matrix.

### Event detection approaches

In a nutshell, EDAs identify dynamic states by grouping time points based on the similarity in the amplitude of BOLD signals (or its derivations) from a subset of the brain’s regions or the entire brain (Figure 2B). The idea behind the EDA can be explained as follows: a group of functionally connected regions (neural assemblies) activates together in response to either internal or external events (stimuli), which results in a momentarily increase in their BOLD signal amplitude. Assuming each dynamic state represents a distinct pattern of spontaneous events, we can obtain brain dynamic states by identifying different (co-)activation patterns (CAPs) in the time series. Note that this is a simplification of the idea behind EDAs and their relationship with the dFC states. The EDAs were initially developed based on the hypothesis that spontaneous BOLD signal originates from infrequent (i.e. sparse in time) neuronal events, such as large-scale neuronal avalanching activity (Chialvo, 2010; Tagliazucchi *et al.*, 2011, 2012). EDAs commonly consist of three steps: (1) detecting the time points in which neural-related events occur, (2) grouping the selected time points to identify different dynamic states and (3) quantifying dynamic properties using different metrics (similar to the examples discussed WBAs).

Several methods have been developed to detect the time points in which spontaneous, infrequent events occur. The simplest and most common category of time point detection methods leverages the amplitude of the BOLD signal. Point process analysis (Tagliazucchi *et al.*, 2012) and CAPs (Liu *et al.*, 2013) commonly use this category of time point detection methods. Two familiar procedures to select time points from the amplitude of BOLD signal are (1) choosing time points which pass a threshold value (e.g. above one standard deviation of the time series) (Tagliazucchi *et al.*, 2011, 2012; Liu and Duyn, 2013) and (2) selecting time points which are the local maxima/minima of the time series (Tagliazucchi *et al.*, 2016). Another category of time point detection methods uses deconvolution techniques, which was previously applied to task-based fMRI studies (Gitelman *et al.*, 2003). A hemodynamic model is used to deconvolve the BOLD signals and resolve them into a set of sparse, event-related time points. Paradigm free mapping (Caballero Gaudes *et al.*, 2013) and total activation (Karahanoglu *et al.*, 2013) are some examples of time point detection methods that use the hemodynamic deconvolution technique. In the same category, the innovation-driven CAPs approach suggests applying temporal derivatives to the deconvolved BOLD signal to capture transient information (identify regions with a simultaneous increase or decrease) (Karahanoglu and Van De Ville, 2015). While standard EDAs are based on the assumption of sparse events, a study may disregard this fundamental assumption and consider all time points for the second step of the analysis (Liu *et al.*, 2013).

In the second step, we divide the selected (event-related) time points into multiple groups based on the similarity between their spatial patterns (commonly using k-means clustering). Each group (cluster) represents a dynamic state and consists of time points with a similar co-activation (spatial) pattern, which is distinct from other groups. Subsequent thresholding is commonly used to identify regions associated with each state. The second step is similar to how WBAs identify dynamic states, but instead of using the similarity between connectivity patterns, EDAs use the similarity between CAPs. Interestingly, the results of EDAs resemble the spatial patterns of well-known FC patterns, such as large-scale networks, which further highlight the similarity between co-activation and FC patterns. This resemblance is somewhat expected as when two regions are co-activated, they are also covarying over time which fits the definition of statistical dependency and FC. Finally, like the third step of WBAs, dynamic properties and the timing of dynamic states can be quantified using various metrics such as dwell time, transition probability and the occurrence rate of dynamic states.

Instead of identifying recurring time points, some EDAs focus on identifying recurring patterns of sequences of time points. In other words, these approaches are interested in finding a particular temporal sequence that repeats over time. For example, quasi-periodic patterns are a sequence of consecutive time points that recur during a scan (Majeed *et al.*, 2011). It is worth mentioning that the idea behind these approaches is closely related to propagating waves observed in other imaging modalities (Matsui *et al.*, 2016; Muller *et al.*, 2018). There are also other approaches that detect dynamic states by characterizing temporal sequence information at the cost of additional computations and stricter assumptions, such as specific state-space models (hidden Markov models) (Eavani *et al.*, 2013; Vidaurre *et al.*, 2017).

While EDAs can be used to evaluate dFC properties, like any other approach, EDA methods come with assumptions and limitations. For instance, the choices of threshold or deconvolution parameters can significantly affect the sensitivity of EDAs to detect the time points of events and therefore alter results. Another major issue is sensitivity to noise. Because the fMRI signal has a low contrast to noise ratio, using individual time points makes EDAs significantly susceptible to noise. As a result, detecting time points of events and allocating time points to dynamic states can be inaccurate due to low SNR. For instance, noise contamination can influence local maxima/minima or alter those time points that survive thresholding, and thus makes the selection of event-related time points inaccurate. Deconvolution techniques are also inherently sensitive to noise and are constrained by the specific assumptions of their HRF model. The susceptibility to noise becomes more concerning when we study the temporal patterns of dFC. For instance, when we use temporal ordering information to identify dynamic states or when we quantify dFC using the temporally dependent measures like dwell time, fraction rate, etc. Moreover, EDAs commonly use anatomical regions as nodes to detect events. This demands additional pre-specified parameters. Using fixed anatomical regions makes EDAs susceptible to functional inhomogeneity within nodes and disregards inter- and intra-subject spatial variability.

## Considerations and caveats

dFC can occur at different time scales, from milliseconds to the entire life span, and fMRI provides an excellent opportunity to non-invasively study whole-brain dynamics in spatial and temporal resolutions simultaneously, which currently cannot be achieved using other imaging modalities. More specifically, fMRI can capture hemodynamic dFC that occurs on the order of seconds with spatial resolution in the order of a millimeter. While the sluggish hemodynamic response limits the upper band of the temporal resolution of dFC captured by fMRI, higher sampling rates and sub-second resolutions have several advantages including: (1) capturing higher-frequency information of dFC; (2) improving specificity and robustness of findings by providing more data for any given temporal scale (e.g. in sliding window); (3) increasing the sensitivity to identify dynamic states and their onset, which is especially critical for EDAs; (4) more accurately quantifying dFC properties (particularly those measuring temporal profile) and (5) improving efficacy of noise-reduction techniques such as reducing the effect of aliasing. While the slow hemodynamic responses are the major contributor to neural-related changes in the fMRI signal, other neural-related properties within higher-frequencies (e.g. 1 > Hz) have also been reported studies (Lin *et al.*, 2015; Lewis *et al.*, 2016). It is thus possible to capture higher temporal resolutions than the conventional 0.01–.15 Hz frequency band (Chen *et al.*, 2017).

dFC is intertwined with many temporal factors such as vigilance, sleep state and arousal states, maturation, aging, and learning experiences (Lurie *et al.*, 2020). While a substantial body of evidence supports the relationship between dFC and neural communication, other mechanisms such as physiology, metabolism, autonomic activities and neurovascular coupling also modulate dFC patterns. These are important factors to consider when we evaluate dFC using the BOLD signal (for prior review see Thompson, 2018). There is a large body of research on what portion (if any) of changes in FC measured in the BOLD signal is related to brain dynamism (Thompson, 2018), and several methods have been developed to evaluate the significance of various FC measurements against different null hypotheses (Bassett *et al.*, 2013; Lindquist *et al.*, 2014; Hindriks *et al.*, 2016; Laumann *et al.*, 2017). However, one should keep in mind any null model only test the presence of a specific type of dFC properties, and the result of a statistical test does not guarantee the presence or absence of dFC (Miller *et al.*, 2018). Hypothesis-driven studies should be conducted to understand the neural basis and mechanisms of dFC estimated by fMRI.

Furthermore, WBAs and EDAs are neither mutually exclusive nor collectively exhaustive as some analytical approaches can be categorized as both and there are approaches that cannot fit into either of these categories. However, WBAs and EDAs are the most established and verified categories which have straightforward applications in clinical and research environments. Other categorizations such as spatially *vs* temporally dynamic approaches, model-based *vs* data-driven techniques and univariate *vs* multivariate analyses can provide a more complete picture of existing approaches.

The BOLD signal is an indirect measurement of neural activities and significantly contaminated with many so-called non-neural signals such as motion, heart rate and respiration. Therefore, it is important to take measures to remove or model the spurious fluctuations and confounding factors from the BOLD signal. At the same time, some of these signals, such as heart rate and motion, are physiological changes associated with neural processes. This impacts the effectiveness of the noise reduction approaches to minimize the contribution of spurious fluctuations, particularly in the absence of ground truth. Therefore, extra care is needed when performing noise-reduction techniques and cleaning procedures to avoid removing meaningful, neural-related information. For instance, although global signal regression, a preprocessing procedure, is shown to improve the relationship between the dFC of BOLD signal and the dynamic changes in simultaneously recorded local field potentials (Thompson *et al.*, 2013b), it may have a negative impact on the reliability dFC analysis (Smith *et al.*, 2018) and cause heterogeneous changes in dFC across the brain (Xu *et al.*, 2018). Regardless of these debates, several pre- and post-processing procedures, including despiking, filtering and nuisance-regression, have been recommended as a trade-off to minimize the impact of spurious fluctuations and statistical uncertainty (Hahamy *et al.*, 2014). However there are still many choices to optimize (e.g. filter early or late, etc.) (Vergara *et al.*, 2017).

While dFC analysis explains specific inconsistencies in sFC findings, the addition of the time-varying properties comes with its own complexities. Furthermore, different analytical approaches use different modeling techniques which might, therefore, capture different aspects of dFC. Some assumptions and limitations of analytical frameworks such as overlooking the inter- and intra-subject spatial variations also lead to inconsistencies and significantly impact the validity of the results. Acquisition parameters and data quality such as low SNR, low temporal resolution or collecting a short segment of data (short scan time) are other components associated with inconsistencies in dFC findings. Furthermore, brain dynamism is unconstrained in nature, and individuals during scans report a variety of different mental activities such as daydreaming, recalling events, planning, dreaming, etc. It is important to consider this when trying to replicate dFN results, for example, it would be unlikely to replicate the same timing for the earlier events; however, this is probably not the ideal goal for a replication study of dFC.

Finally, while task-based dFC is relatively young within this field, it has high potential. Studies demonstrated that task-modulated dFC (dFC during a task) can predict task performance. In addition, pre-stimuli dFC can predict responses to upcoming events (for review, see Gonzalez-Castillo and Bandettini, (2018)). However, task-based dFC studies carry additional challenges compared to resting dFC analyses. For instance, a task-related BOLD signal can generate CAPs arguably not related to intrinsic dFC. Thus, it is common to remove task-induced activity before computing dFC. However, given within- and between-subject variability and the possibility of task effects being modulated by or modulating intrinsic connectivity, the efficiency of this procedure is under question. Examples of subject-specific variability include differences in response to tasks, temporal profiles of neural activity and hemodynamic responses. Incorrect implementation of this procedure will not only fail to remove task-induced co-activation, but it may also induce spurious fluctuations in dFC. One approach is to regress out both the averages of task-induced activity and its first derivative to account for transient task-induced activity) (Gonzalez-Castillo and Bandettini, 2018). In contrast to removal, other approaches consider task-modulation within the context of intrinsic connectivity. One procedure is to evaluate the relationship between dFC and ‘task-load function’ (Sakoglu *et al.*, 2010). The task-load function is a measure of a subject engagement with a given task over time, and in WBAs, it can be computed as the time-windowed integral of the HRF-convolved task paradigm. The correlation between the windowed-FC during a task and the task-load function is a straightforward, intuitive way to implement task-concurrent dFC (Sakoglu *et al.*, 2010). This approach has been implemented in the GIFT toolbox (see ‘Implementing a dFC study: a GIFT walkthrough’), and further details on the implementation of task-based dFC can be found at Gonzalez-Castillo and Bandettini, (2018).

## Concluding remarks

dFC analysis using fMRI is still a very active area of development, but it is quickly turning into a critical element of brain research because it provides exceptional opportunity to study brain dynamism and its relationship with different mental states, cognitive conditions and disorders. In this manuscript, we review some research on the potential association of fMRI-dFC with cognitive demands and behavioral performance. We also present examples of how dFC patterns are disrupted in various brain disorders and the relationship of atypical dFC patterns with both cognitive impairments and the outcomes of the disorders (for reviews, see Calhoun and Adali (2016), Keilholz *et al.* (2017), Cohen (2018), Thompson (2018), Lurie *et al.* (2020)). Alterations in dFC patterns across a wide range of conditions have been observed even in the absence of sFC differences. One proposition is that each sFC pattern is an average of a set of dFC patterns and therefore smooths out the nuanced differences (Iraji *et al.*, 2020). The dFC patterns are capable of encoding variations across conditions that are more transient than those captured by conventional sFC analyses. Considering the dynamic nature of the brain, the dFC information obtained from fMRI might be a crucial piece in providing a more thorough understanding of the brain’s function and characterizing neurological and psychiatric disorders. Quantifying the spatial and temporal dynamics of the brain opens more opportunities to study the brain through a window of dynamism and help answer some of the most compelling questions in cognitive and affective neuroscience. However, this goal requires a hypothesis-driven, carefully designed study and contributions from neuroscientists. Moving forward, dFC research benefits from careful design studies, which allows researchers to understand the mechanisms underlying dynamism, identify neural etiology, elucidate the mechanisms of healthy cognition, investigate individual differences in cognition, and probe dFC alterations and disruptions in the brain illnesses. This can potentially lead to identifying imaging-based clinical biomarkers for early diagnosis and disease treatments. We encourage researchers who are new to dFC analysis to become more familiar with different analytical steps and limitations of techniques in each step. We also emphasize the importance of inter- and intra-subject variability and the need to consider spatial dynamic properties in future studies (Iraji *et al.*, 2020).

## Implementing a dFC study: a GIFT walkthrough

In this section, we provide a brief overview of the dFC options available in the GIFT toolbox and provide a quick walkthrough to facilitate the application of dFNC analyses for those new to this field. The GIFT toolbox and manual can be found at https://trendscenter.org/software/gift/. GIFT is a MATLAB toolbox which can be used standalone (i.e. a complied version without a MATLAB license) or with MATLAB. It also includes a python interface (giftpy) and is also released as a (docker) containerized tool which also does not require a MATLAB license. GIFT consists of a wide range of ICA, allowing group inferences from the fMRI data. It also contains several other toolboxes, such as the dFC toolbox to study brain dynamism and the Mancovan toolbox to determine the significant covariates using multivariate analysis of covariance and a stepwise regression model as well as univariate testing. The resulting covariates found out to be significant can be used in a univariate framework. The noise cloud toolbox uses both spatial and temporal characteristics of independent components to automatically identify noise/artifact components from the specified components after an initial training process. This can also be used to clean fMRI data. To clean data, we can use the remove component(s) option within the GIFT toolbox. ICA is a powerful method for cleaning fMRI data prior to dynamic and static FC studies, including for ROI-based analysis. In addition, we developed SimTB (A simulation toolbox for fMRI data) toolbox to generate simulate data and to test different analytical methods. SimTB gives users full control over generating data, including the creation of desirable spatial patterns of sources, the implementation of block-related and event-related experimental designs, the inclusion of tissue-specific baselines and simulated head movement.

**Fig. 3.**
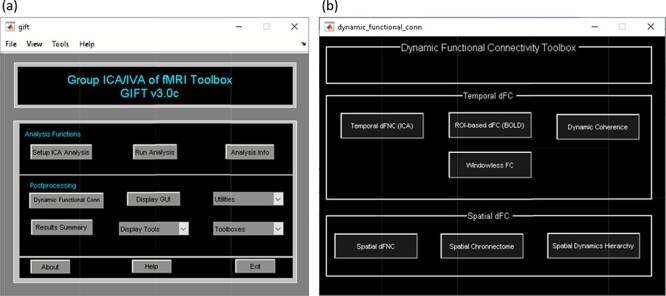
Left: GIFT toolbox. Right: dynamic functional connectivity toolbox.

A video tutorial of the dFC pipeline is available at https://trendscenter.org/software/gift/videos. After adding the GIFT directory to the MATLAB search path, the GIFT toolbox can be launched by entering ‘gift’ into the MATLAB command line. By clicking on the dFC button, the ‘dynamic functional connectivity’ toolbox appears and allows you to choose among several available dFC techniques (Figure 3). The dFC techniques are separated into two categories, temporal dynamics and spatial dynamics.

Temporal dynamic analyses (‘Temporal dFC’ options) study dFC via variations in the temporal patterns of sources, which are commonly achieved by studying the changes in their statistical dependency over time (Iraji *et al.*, 2020). Spatial dynamic analyses (‘Spatial dFC’ options) focus on variations in the spatial patterns of sources over time (Iraji *et al.*, 2020) (Figure 3). The list of measures available in GIFT to quantify dFC properties can be found in Table 1.

### Temporal dFC

#### Temporal dFNC toolbox.

**Fig. 4.**
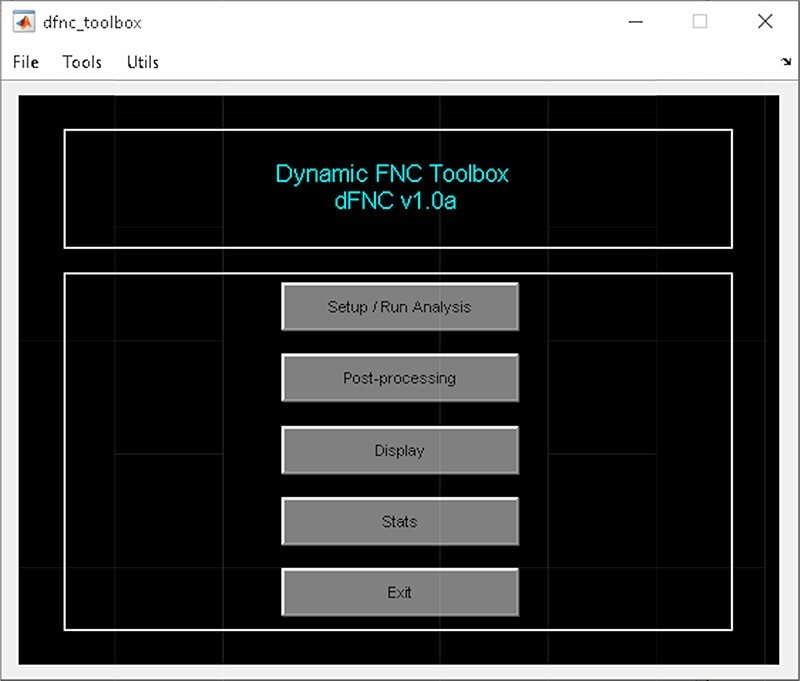
Temporal dFNC toolbox.

**Fig. 5.**
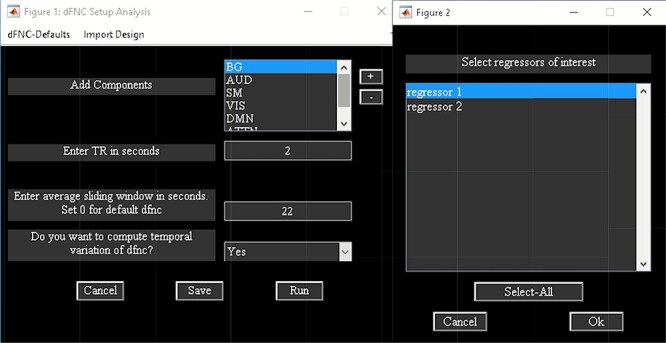
Temporal dFNC setup analysis.

**Fig. 6.**
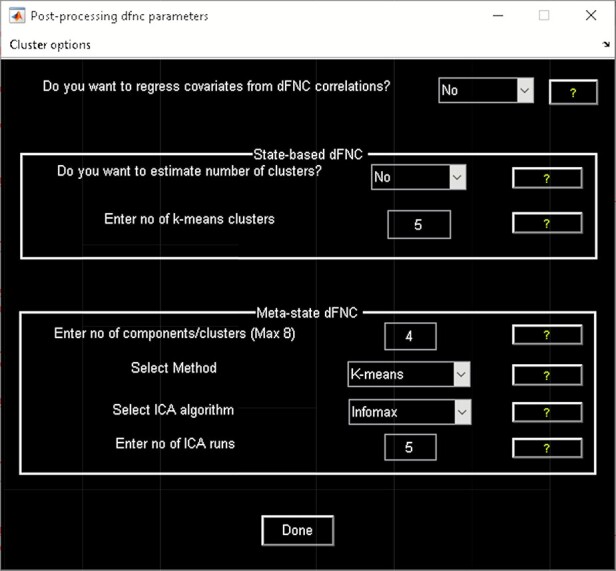
Post-processing dFNC.

**Fig. 7.**
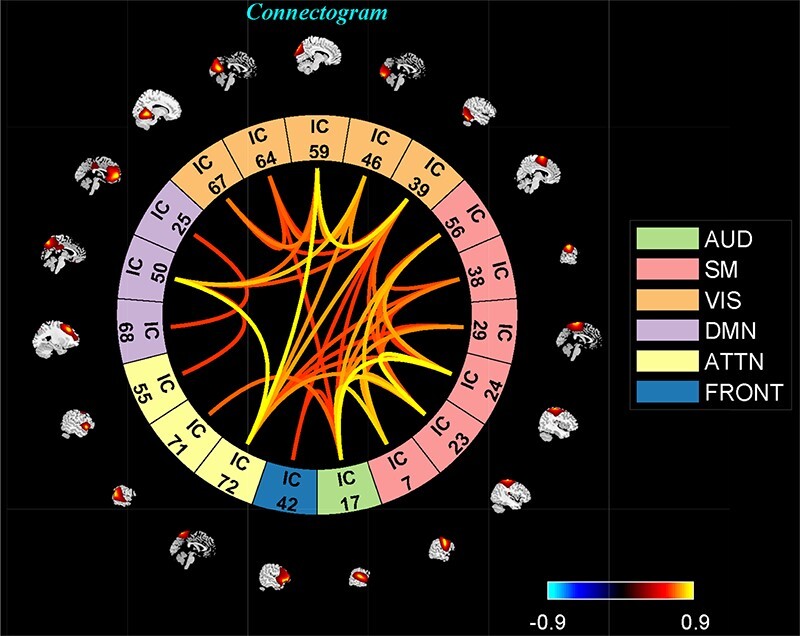
One of the clusters represented as a connectogram.

It is a WBA which computes whole-brain dFC (Allen *et al.*, 2014). It uses ICA from the GIFT toolbox to estimate nodes/sources and their associated time course. To access temporal dFNC, click on ‘Temporal dFNC (ICA)’ button under Temporal dFC options (Figure 3). Briefly, the steps include the following (Figure 4):

1. ‘Setup/Run Analysis’ panel (Figure 5) in which we enter the repetition time (TR) of the experiment and organize components by functional domains/networks which will be useful in plotting FNC matrices at the end of the analysis. dFNC defaults menu allows users to choose different preprocessing options and dFC parameters. For preprocessing, options include detrending, despiking, low-pass/band-pass filtering and regressing out confounding covariates from time courses. dFNC parameters include the parameters associated with window (e.g. window size, the alpha parameter of the Gaussian window) and the regularization method (L1 or none). After completing the parameter selection, use the Run button to run the dynamic FNC. Windowed dFNC matrices are saved for each subject.
2. ‘Post-processing’ option (Figure 6) which consists of two panels: state-based dFNC and meta-state dFNC (meta- state analysis). For state-based analysis, we can enter the number of *k*-means clusters (states) or estimate it using various algorithms, such as Gap statistic, Akaike/Bayesian information criteria, Dunns Index and Silhouette algorithms We can also custo- mize *k*-means options like the number of *k*-means iterations, the distance metric, the maximum number of iterations and the number of reference datasets used for the gap statistic in ‘Cluster options’ menu. For meta-state analysis, several methods like *k*-means, PCA and various ICA techniques are available. It should be noted that ‘Post-processing’ options are similar across different dFC analyses (some have already been implemented in GIFT); therefore, for the sake of brevity, we will not repeat them in the other techniques.
3. ‘Display’ option allows us to visualize the result of both (meta-) state results, such as state dFNC, connectivity patterns of meta-state dFNC and connectogrom plot (e.g. Figure 7).
4. ‘Stats’ option provides various statistical analysis choices, like one sample *t*-test, two-sample *t*-test and paired *t*-test on the dFNC strength of each state and (meta-) states metrics.

Temporal dFNC Toolbox also consists of ‘Task-based dFNC’ option (Sakoglu *et al.*, 2010), which uses experimental design information (regressors) as input. Regressors are obtained by convolving onsets with a hemodynamic response function. To compute task-based dFNC, click on the ‘Import Design’ menu available in the ‘Setup/Run Analysis’ panel (Figure 5). The sliding window approach is applied to the task-based regressors and ICA components’ time courses. Correlation is computed between the windowed-FC and windowed task-load function. Task load function is obtained by computing the average of the model time courses at each window (Sakoglu *et al.*, 2010). Options are provided to apply statistical testing to the correlations.

#### Temporal dFC (BOLD) toolbox.

In contrast to temporal dFNC, ‘Temporal dFC (BOLD)’ uses predefined regions of interest (ROIs), and the average BOLD signals within ROIs are used to calculate dFC patterns at each window. It should be noted that we do not recommend using predefined ROIs to study dFC as they do not consider inter- and intra-subject variation. Two options are available in this toolbox. The first one is ROI-ROI dFC in which the user inputs ROIs mask, and WBA is used to estimate dFC between ROIs. The other option is to estimate ROI-to-voxel dFC in which windowed-FC is calculated between the average BOLD signal of each ROI and the BOLD signal of every voxel in the brain. After calculating windowed-FC, the rest of the analysis would be similar to temporal dFC analysis. For instance, *k*-mean clustering can be used to estimate dFC states, and we can perform meta-state analysis in the same manner.

#### Dynamic coherence toolbox.

**Fig. 8.**
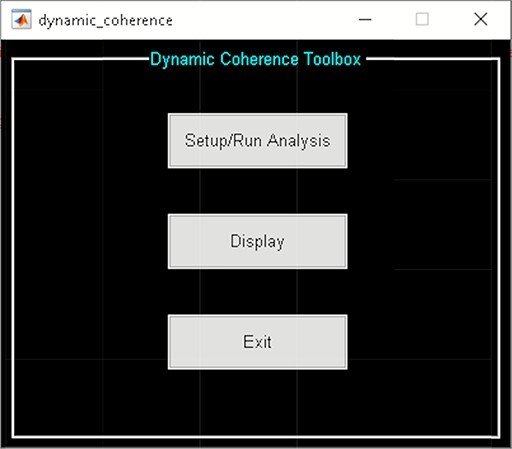
Dynamic coherence toolbox.

**Fig. 9.**
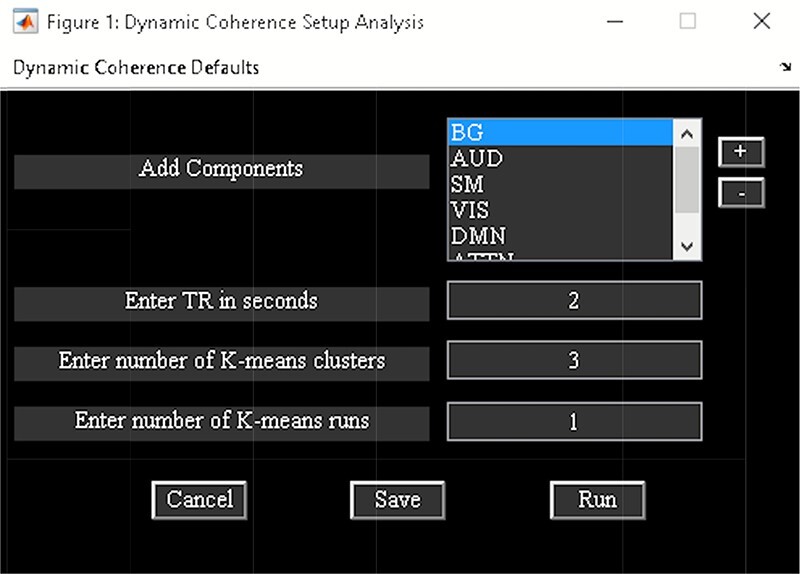
Dynamic coherence setup analysis.

**Fig. 10.**
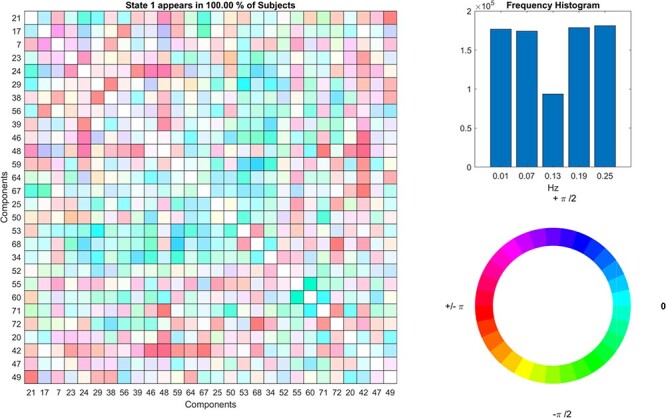
Dynamic coherence results.

‘Dynamic Coherence’ applies complex Morlet wavelet on the time courses of ICNs to capture dFNC in the augmented time and frequency space (Yaesoubi *et al.*, 2015a). In other words, it estimates dFC at different frequencies and phase lags. Dynamic Coherence Toolbox is divided into two parts (Figure 8):

1. ‘Setup/Run Analysis’ allows us to enter analysis parameters. Options are provided to group components by network names and to enter experimental TR in seconds and complex *k*-means specific setting like the number of clusters and *k*-means replicates (Figure 9). There are options to preprocess the time courses like detrending, despiking, filtering and regressing out variance associated with noise from the time courses when you click the ‘Dynamic Coherence Defaults’ menu. After the analysis is complete, the cluster states information is saved to the disk.
2. ‘Display’ option visualizes the results of the analysis, including estimated dFC states and associated frequency and phase histogram (e.g. Figure 10).

#### Windowless FC toolbox.

**Fig. 11.**
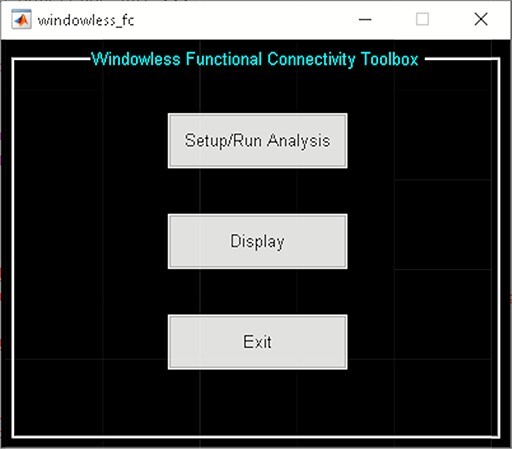
Windowless FC toolbox.

**Fig. 12.**
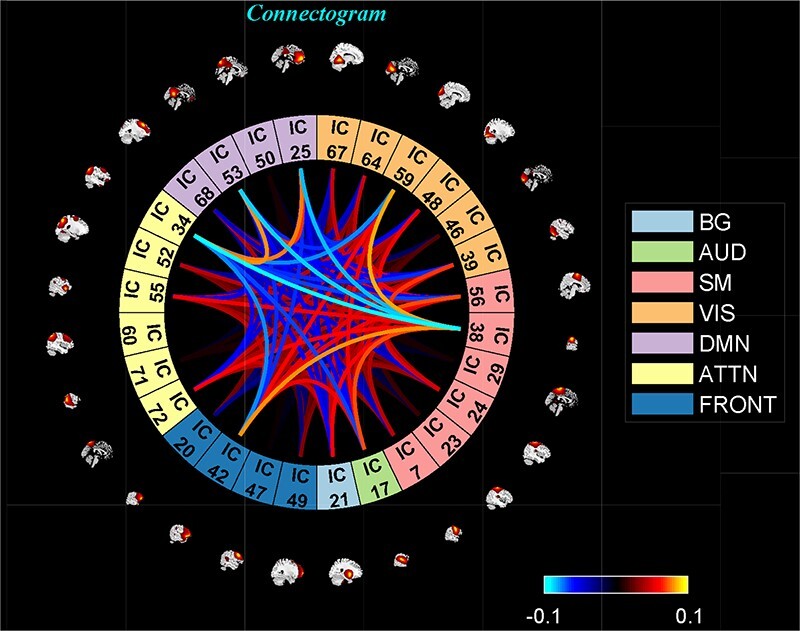
An example of one (K-SVD) dFC state.

‘Windowless FC’ bypasses windowing operation by directly measuring linear dependence in the sample space (Yaesoubi *et al.*, 2018). This approach calculates dFNC states as the outer product between the subspace bases estimated using K-SVD. As a result, it can detect dFC patterns with arbitrary rates of changes. The toolbox consists of two panels (Figure 11):

1. ‘Setup/Run Analysis’ panel provides options for preprocessing the time courses of ICNs, selecting the number of dictionary elements, and the maximum number of iterations. After the analysis, dictionary elements and mixing coefficients are saved to the disk space.
2. ‘Display’ panel allows users to organize components by networks for displaying purposes using matrix plots or a connectogram (e.g. Figure 12).

### Spatial dFC

#### Spatial dFNC toolbox.

**Fig. 13.**
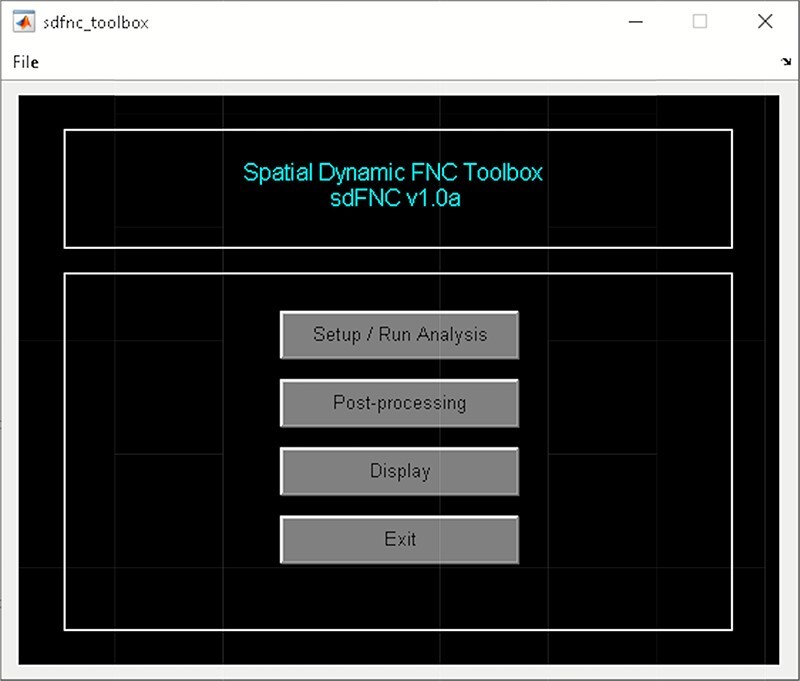
Spatial dFNC toolbox.

**Fig. 14.**
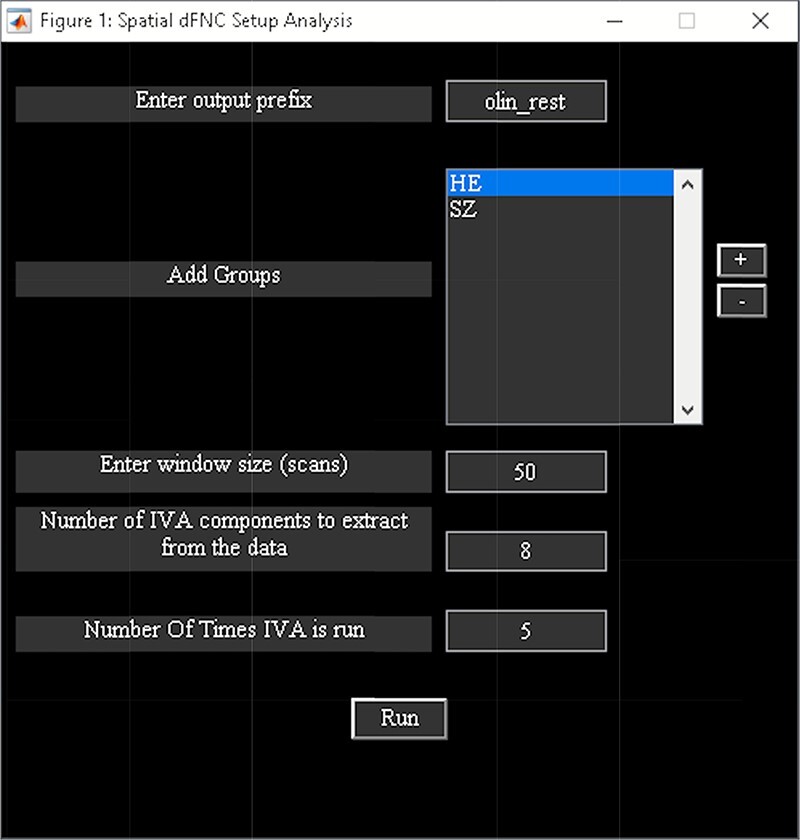
Spatial dFNC setup analysis.

**Fig. 15.**
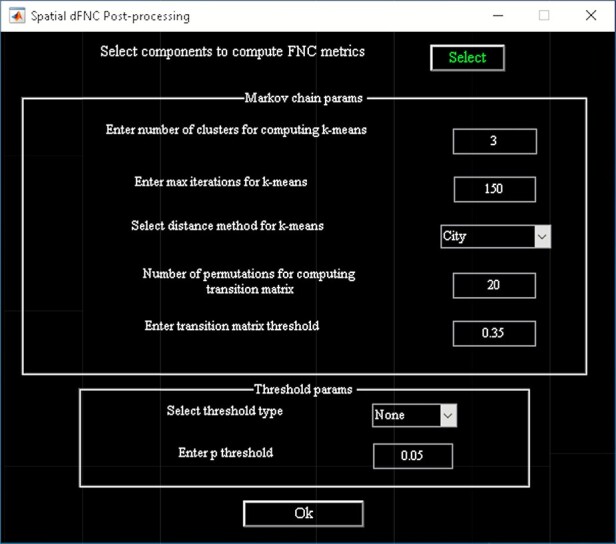
Spatial dFNC post-processing graphical user interface (GUI).

**Fig. 16.**
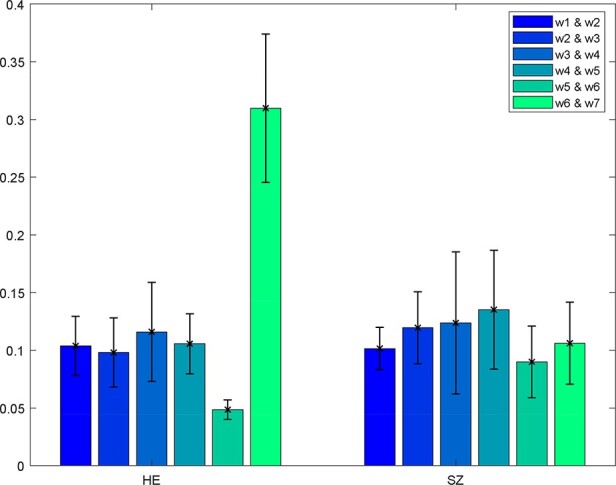
One example of the spatial dFNC results. Kullback–Leibler divergence is computed between pairs of windows.

‘Spatial dFNC’ treats data from each time window as a separate dataset for independent vector analysis (IVA) to capture variations in the spatial pattern of each source over time (Ma *et al.*, 2014). The steps of spatial dFNC include (Figure 13):

1. ‘Setup/Run Analysis’ in which we enter the window size, the number of IVA components and the number of IVA run (Figure 14). We also assign subjects into different groups in this panel (Figure 14).
2. ‘Post-processing’ in which we insert the parameters of Markov chain analysis and the threshold for *t*-tests (Figure 15).
3. ‘Display’ in which all the spatial dFNC results (e.g. Figure 16) are summarized in an HTML page and shown in a web browser.

#### Spatial chronnectome toolbox.

‘Spatial Chronnectome’ captures on voxel-wise changes in the spatial patterns of sources over time. While the original work (Iraji *et al.*, 2019a) used pair-wise correlation to calculate the association of each voxel to a given source/network regardless of its contribution to other sources, the partial correlation is also implemented in GIFT to evaluate the spatial dynamics of each source while controlling for the contribution of other sources. The toolbox uses ICA results from the GIFT toolbox to select the source of interest and their associated time courses.

**Fig. 17.**
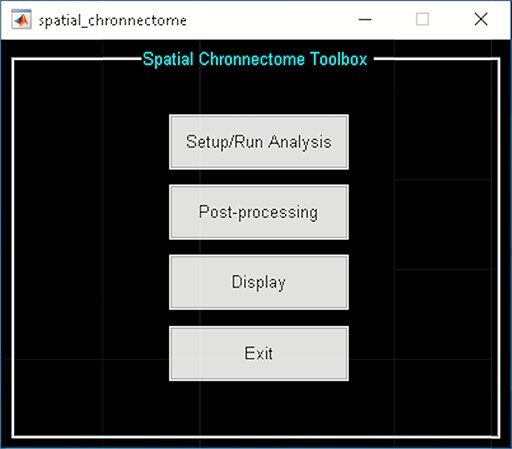
Spatial chronnectome toolbox.

**Fig. 18.**
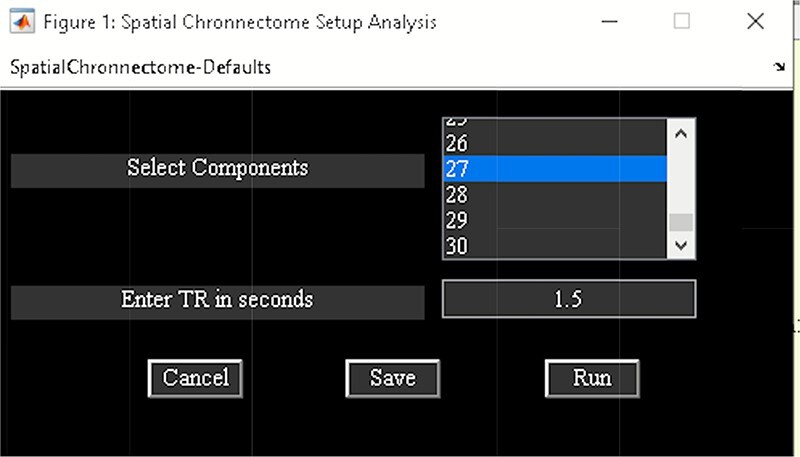
Spatial chronnectome setup analysis.

**Fig. 19.**
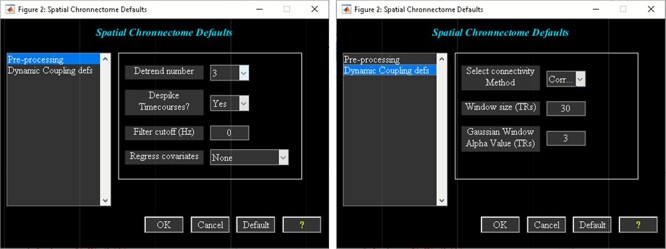
Spatial chronnectome defaults menu. Left: preprocessing options; Right: dynamic coupling prefs options.

**Fig. 20.**
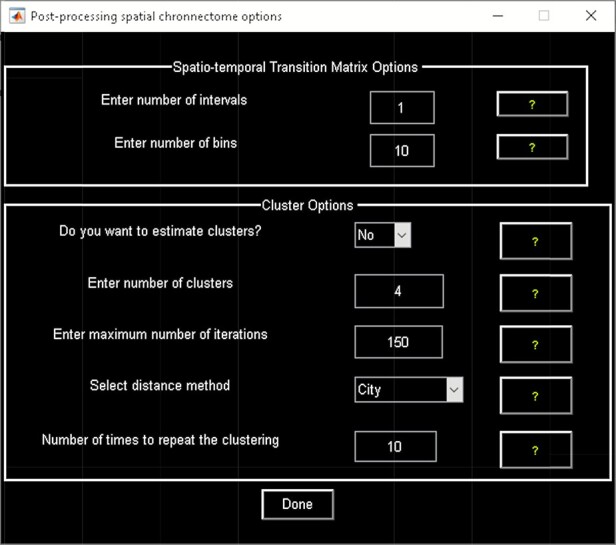
Spatial chronnectome post-processing.

**Fig. 21.**
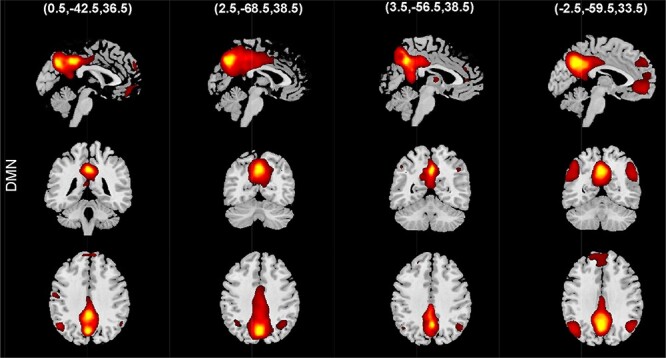
An example of spatial chronnectome results. States are presented as vertically stacked orthogonal slices.

The toolbox is divided into three steps (Figure 17):

1. ‘Setup/Run Analysis’ in which we can enter the analysis parameters (Figure 18). When you click on Setup/Run, a figure window will open to select sources (ICA components) of interests and to enter experimental TR in seconds. ‘Spatial Chronnectome Defaults’ menu contains options for preprocessing BOLD and ICA time courses as well as options for computing dynamic coupling maps in the ‘Dynamic Coupling Prefs’ entry (Figure 19). BOLD signal and ICA components’ time courses are preprocessed (despiking, filtering and motion covariates variance removal) before calculating dFC maps. The parameters for sliding window procedures can be entered in ‘Dynamic Coupling Prefs’.
2. ‘Post-processing’ (Figure 20) in which we quantify the dFC properties. This step calculates the coupling variability map, spatiotemporal transition matrix and spatial states associated with each source (network) and their properties such as dwell time, occupancy rate and transition matrix. Parameters of *k*-means clustering like the number of clusters, the number of max iterations and the distance metric can be selected here. We can also estimate the number of clusters using various algorithms, such as the gap statistic, Akaike/Bayesian information criteria, Dunns index and Silhouette algorithms, available in the ‘cluster options’ panel. A recent study compares a number of cluster validation indices (Vergara *et al.*, 2020).
3. ‘Display’ in which the results of spatial chronnectome analysis for each source of interest will be displayed (e.g. Figure 21).

#### Spatial dynamic hierarchy toolbox.

**Fig. 22.**
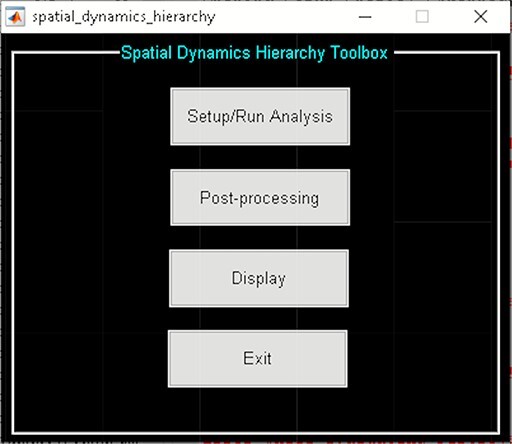
Spatial dynamics hierarchy toolbox.

**Fig. 23.**
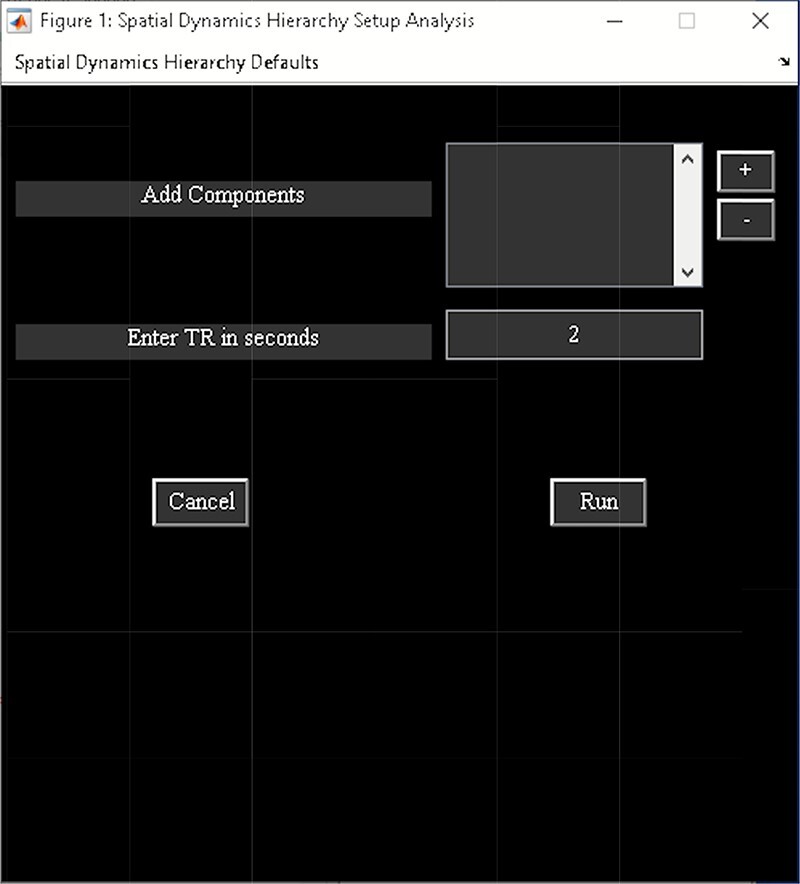
Spatial dynamics hierarchy setup analysis.

**Fig. 24.**
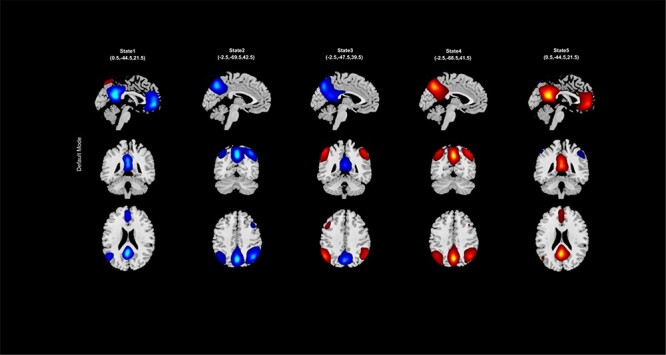
An example of spatial states for the default mode.

The spatial dynamic hierarchy model studies the dynamic properties within brain hierarchy models. In the current version of GIFT (Version 4.0c), the method presented in (Iraji *et al.*, 2019b) assumes fixed membership assignments between the elements of a hierarchy model and captures spatial dynamics within the functional domain as well as temporal dynamics within and between functional domains. Future versions will allow changes in membership assignments over time. This Toolbox is divided into three parts (Figure 22):

1. ‘Setup/Run Analysis’ panel in which we enter the analysis parameters (Figure 23). We assign the components to functional domains and choose the parameters of clustering. Like other toolboxes, we can estimate the number of clusters using various algorithms, available in the ‘cluster options’ menu. We can also choose parameters for preprocessing or cleaning ICA components’ time courses, including despiking, low pass or bandpass filtering and regressing out covariates.
2. ‘Post-processing’ in which we quantify the dFC properties. This step calculates dFC states associated with each functional domain and their properties such as dwell time, occupancy rate and transition matrix. It also computes Functional State Connectivity and associated Functional modules.
3. ‘Display’ in which the results of spatial dynamic hierarchy analysis, such as states associated with each functional domain, are displayed (e.g. Figure 24).

It worth mentioning that we have developed other toolboxes to facilitate the advancement of neuroimaging research. For instance, the fusion ICA toolbox contains several analytical techniques, such as joint ICA (Calhoun *et al.*, 2006), parallel ICA (Liu *et al.*, 2009) and CCA-Joint ICA (Sui *et al.*, 2009), multi-set canonical correlation analysis (MCCA) (Adali *et al.*, 2015), transposed independent vector analysis (IVA) (Adali *et al.*, 2015), Parallel-Group ICA + ICA (PGICA) (Qi *et al.*, 2019) and deep fusion to analyze multimodal data (Plis *et al.*, 2018). Joint ICA can be applied to different modalities (or task-fMRI) to extract maximally spatially independent maps for each modality (or task) that are coupled together by a shared loading parameter (in other words mixing coefficients is fixed between the modalities) (Calhoun *et al.*, 2006). Parallel ICA is an extension of ICA that allows simultaneously running ICA on multiple modalities (Liu *et al.*, 2009). For example, for two modalities, it extracts sources from both modalities and connections between them. Comparing with Joint ICA, where a shared mixing matrix is used for both modalities, parallel ICA assumes the two data sets are mixed in a similar pattern but not with identical parameters. CCA + joint ICA uses canonical correlation analysis (CCA) and ICA to extract both shared and distinct sources across features and mixing coefficients (Sui *et al.*, 2009). MCCA estimates sources using the similarity in the mixing coefficients between the different modalities (Adali *et al.*, 2015). Transposed independent vector analysis incorporates higher-order statistics in the MCCA model to extract common features across modalities (Adali *et al.*, 2015). PGICA uses temporal information from first-level group ICA into a Parallel ICA framework. PGICA can detect linked FNC and structural covariations in the components when first-level fMRI and structural MRI (sMRI) datasets are used (Qi *et al.*, 2019). Deep Fusion uses neural networks for finding associations between fMRI and sMRI datasets (Plis *et al.*, 2018). Collaborative Informatics and Neuroimaging Suite Toolkit for Anonymous Computation (COINSTAC) is another useful toolbox that was designed to address the need for sharing and collaboration in effective and easy-to-use manner. COINSTAC provides tools to perform decentralized, privacy-enabled analysis to eliminate the need of directly sharing the data (Plis *et al.*, 2016). We have implemented a number of algorithms within COINSTAC including preprocessing for fMRI, sMRI, diffusion MRI data as well as regression, group ICA, dynamic connectivity, support vector machine classification and many more.
